# DNMTs and Impact of CpG Content, Transcription Factors, Consensus Motifs, lncRNAs, and Histone Marks on DNA Methylation

**DOI:** 10.3390/genes11111336

**Published:** 2020-11-12

**Authors:** Jaqueline Loaeza-Loaeza, Adriana S. Beltran, Daniel Hernández-Sotelo

**Affiliations:** 1 Laboratorio de Epigenética del Cáncer, Facultad de Ciencias Químico-Biológicas, Universidad Autónoma de Guerrero, NC 39087 Chilpancingo, Mexico; jaquelineloaeza@uagro.mx; 2 Department of Pharmacology, University of North Carolina, Chapel Hill, NC 27599, USA; adriana_beltran@med.unc.edu

**Keywords:** DNMT, methylation, consensus motifs, CpG content, histone marks and transcription factors

## Abstract

DNA methyltransferases (DNMTs) play an essential role in DNA methylation and transcriptional regulation in the genome. DNMTs, along with other poorly studied elements, modulate the dynamic DNA methylation patterns of embryonic and adult cells. We summarize the current knowledge on the molecular mechanism of DNMTs’ functional targeting to maintain genome-wide DNA methylation patterns. We focus on DNMTs’ intrinsic characteristics, transcriptional regulation, and post-transcriptional modifications. Furthermore, we focus special attention on the DNMTs’ specificity for target sites, including key *cis*-regulatory factors such as CpG content, common motifs, transcription factors (TF) binding sites, lncRNAs, and histone marks to regulate DNA methylation. We also review how complexes of DNMTs/TFs or DNMTs/lncRNAs are involved in DNA methylation in specific genome regions. Understanding these processes is essential because the spatiotemporal regulation of DNA methylation modulates gene expression in health and disease.

## 1. Introduction

An interesting paradigm in cell fate is to understand how different cellular lineages originate from a single cell precursor and the same genome. One characteristic of the specific cell type is a particular profiling expression encoded in its genome. In mammals, DNA-based processes are highly regulated by epigenetic mechanisms that impact biology and chromatin transcriptional states. Epigenetic studies include, but are not restricted to, heritable modifications on chromatin, independent of alterations in the DNA sequence. Epigenetics involves DNA and RNA methylation, post-translational histone modifications, transcriptional regulation by long noncoding RNAs (lncRNAs), and physicals alterations in nucleosomal positioning. Epigenetic mechanisms are composed of regulation layers set through writers, readers, and epigenetics erasers that allow the dynamic cell-specific gene expression [1,2]. Epigenetics contributes to cell plasticity modeling by establishing several expression profiles and, therefore, distinct phenotypes.

DNA methylation is one of the best-characterized epigenetic modifications, and it has a critical role in active and inactive chromatin equilibrium for gene expression control [3]. Gene silencing by DNA methylation is necessary to balance and regulate cell biological processes. DNA methylation is a chemical modification that occurs in the carbon 5 of cytosines located at the 5′ of guanine, and this arrangement is called CpG dinucleotide [4]. DNA methylation augments the information contained in the DNA sequence, and confers read duality to the same sequence, changing the functional status of a gene from active to inactive or vice versa [1,3]. DNA methylation is deposited on a CpG context by DNA methyltransferases (DNMTs). DNMTs are a family of mammalian enzymes that convert cytosine to 5-methylcytosine (5mC). In humans, there are three catalytically active DNMTs: DNMT1, DNMT3A, and DNMT3B. DNMT1 is responsible for maintaining existing methylation, while DNMT3A and DNMT3B establish dynamic methylation patterns. These enzymes are finely regulated, and deregulation of their expression leads to abnormal methylation [5,6]. The strict regulation of genes via methylation ranges from specific allele methylation patterns to differentially expressed genes between stem cells and adult cells. Behind the DNMTs activity and DNA methylation deposition, there are key mechanisms through *cis*- and *trans*-regulatory factors that influence DNA methylation. *Cis*-regulatory elements (CREs) form a key class of regulatory noncoding DNA sequences, which act to regulate transcription of a neighboring gene. Structurally, the genome is segmented into biologically active *cis* elements, such as enhancers, insulators, locus control region, promoters, CpG islands (CGIs), and binding sites for transcription factors [7], which can be methylated as a second regulatory level [8]. Moreover, *trans*-regulatory proteins act through two domains, first by binding to a particular DNA domain (Zn fingers, Leu zipper), and the second is responsible for activity on transcription (as rich in Gln, rich in Pro, acidic α-helix domain) [9,10]. In the human genome, DNA methylation is spread in around 28 million CpG dinucleotides distributed in more than half of the genes and can be directed by *cis* and *trans* elements. CpG dinucleotides are commonly condensed in regions called CpG islands that are short interspersed DNA sequences with an average size of 500 to 1000 bp and >60% CpG content relative to the bulk genome [11]. CGIs are frequently found in the regulatory region of about 72% [12] of protein-coding genes and are known to impact their transcriptional regulation by modulating the accessibility and affinity of transcription factors to their binding sites [13,14]. The promoter regions and CGIs from methylated genes also contain smaller common sequences that are short DNA motifs. These sequences can promote the affinity of DNMTs by an unknown mechanism. These motifs are found in DNMTs’ enrichment peak and suggest that they can be binding motifs for proteins or other molecules that regulate DNA methylation [15]. The DNA methylation landscape is molded by other epigenetics modifiers such as long noncoding RNAs (lncRNAs) and histone modifications. LncRNAs are RNAs with a length of more than 200 nucleotides and do not encode proteins but can interact with DNMTs and localize them to specific genes [16,17]. Histone modifications contribute to the regulation of gene expression and are associated with DNMTs’ function in several genes. Histones are central components of chromatin, and the residues of histone tail may undergo post-translational modifications such as phosphorylation, acetylation, and methylation. The complex dynamic combination of these histone marks regulates active or repressive chromatin states. In particular, mono-, di-, and trimethylation of lysines is associated with positive or negative chromatin methylation landscapes [18,19]. In this review, we integrate information concerning DNMTs and regulatory elements associated with the deposition of DNA methylation in genes targeted by the DNMTs in mammalian genomes.

## 2. Discovery of 5-Methylcytosine and Its Function in the Genome

**Figure 1:**
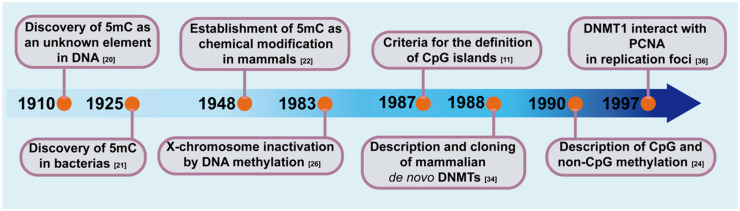
Timeline of key findings that led to the discovery and role definition DNA methyltransferases (DNMTs) in mammalian genomes and gene expression.

In vitro, in early studies on the composition and biochemical properties of nucleic acids, 5mC was discovered, and it was described as an unknown element in the DNA [20]. Later, while differences between pathogenic and nonpathogenic bacteria were being searched for, 5mC was confirmed as a product of the hydrolysis of *Bacillus tuberculosis* nucleic acids and was identified as the fourth pyrimidine (cytosine, thymine, uracil, and 5-methylcytosine) [20,21]. Consequently, 5mC was identified in mammalian DNA and was described with acid–alkaline properties similar to cytosine, but without being uracil [22]. Almost parallel to the reports of 5mC, in 1939, Waddington proposed the existence of an epigenotype to explain certain aspects of development that were influenced by the environment and not the result of changes in the genotype [23]. Functional studies of DNA methylation evolved from a protective mechanism against foreign DNA in bacteria to a regulatory mechanism of gene expression in vertebrates [24]. Studies in *Escherichia coli* demonstrated that when foreign DNA was not digested by endonucleases, the DNA was methylated as an alternative host protection mechanism [25]. Based on prokaryotic observations regarding the presence of enzymes that methylate DNA and after visualization of problems with the existent models, Rigss et al., intuited the existence of mammals’ DNMTs and consequently raised the X-chromosome inactivation model via DNA methylation [26]. From studies in both vertebrates and plants [27] emerged the first hint of 5mC in gene regulation in 1975 [28]. The direct correlation between gene repression and differentiation was established in 1980. Globin genes were analyzed by means of restriction endonucleases, which are unable to cleave methylated DNA (*HpaII*). In the germ line, all sites tested in the globin gene region were methylated; however, in somatic tissue, DNA methylation was absent at specific sites in the globin gene [29]. Additionally, the treatment with Cytidine analog (5-azacytidine) in mouse embryo cells induced changes in the differentiation as a consequence of methylation inhibition of newly synthesized DNA [30]. At this point, the aforementioned assays confirmed the existence of specific methylation patterns, symmetric in both chains, heritable, and tissue-specific [31]. Gruenbaum and Bestor and Ingram worked on the pioneering studies of vertebrate DNMTs. These studies included substrate-dependent DNA methylation that showed DNMTs preference for hemimethylated CpG sequences, and that later was established as their methylation mechanism and function [4,29]. DNMTs were sequenced and cloned from mouse cells to study their functional domains and decipher their action mechanism [30]. DNMTs and DNA methylation was established as an integral component of the gene expression regulation in mammals (Figure 1). The global DNA methylation patterns in mammals are established by three enzymatically active DNA-methyltransferases: DNMT1, DNMT3A, and DNMT3B.

## 3. DNA Methyltransferases (DNMTs)

### 3.1. Characteristics and Function of DNMT1 (Maintenance Methylation)

DNMT1 maintains current methylation patterns in the DNA, and it is an abundant enzyme in somatic cells. It is highly conserved in eukaryotes and constitutively expressed in dividing cells. DNMT1 consists of 1620 amino acids and 10 conserved motifs involved with its catalytic function [32]. It has a large N-terminal domain with regulatory function and a smaller C-terminal catalytic domain related to DNA methyltransferase activity. The N-terminal domain regulates the recognition of methylation target sites through several subdomains, such as DNA methyltransferase-associated protein 1 (DMAP), the PCNA (proliferating cell nuclear antigen) binding domain (PBD), the replication foci-targeting sequence (RFTD), C, cysteine; X, any amino acid (CXXC), and Bromo-adjacent homology 1 and 2 domains (BAH1 and BAH2) [33]. Each domain functions in a specific manner. For example, the CXXC domain acts as a sensor for unmethylated CpG [34]; DMAP motif is essential for the recruitment of the transcriptional repressors as DMAP1 and HDACs in the replication foci [35]; the PBD motif interacts with PCNA [36]; and RFTDs that interact with Ubiquitin-like with PHD and Ring finger domains 1 (UHRF1) to recruit DNMT1 at the DNA replication site and localizes DNMT1 to the centromeric chromatin and replication foci [37]. DNMT1 is more abundant during the entrance to the S phase and it is responsible for symmetrically adding the methyl group to the CpGs [6,38]. DNMT1 activity is structurally dependent on a hemimethylated substrate [39]. DNMT1 adds methyl groups to the unmethylated cytosines at the nascent chain to generate symmetric methylation. The active center of the C-terminal domain interacts specifically with a preference 30–40-fold higher for hemimethylated DNA [34,40,41]. DNMT1 knockout in mice revealed that it is required for appropriate embryonic development, genomic imprinting, and X-chromosome inactivation [42,43].

### 3.2. Characteristics and Function of DNMT3A and DNMT3B (De Novo Methylation)

DNMT3A and DNMT3B enzymes are essential for de novo methylation during early development as their knockouts are lethal during embryogenesis in murine models [44,45]. DNMT3A and DNMT3B display a high degree of similarity, especially in their catalytic domain, in which they share about 84% homology [46]. However, despite this, they have different methylation mechanisms and nonredundant functions. DNMT3A has a cooperative methylation mechanism, whereas DNMT3B methylated DNA by a noncooperative mechanism [47,48]. DNMT3A is found on the small arm of chromosome 2 at position 23.3 and consists of 26 exons/25 introns and codes for 912 amino acids. DNMT3B is found on the long arm of chromosome 20 at position 11.21, 24 exons/23 introns, and its genome codes for a protein of 853 amino acids. DNMT3 enzymes contain a variable N-terminal portion of 280 and 220 amino acids for DNMT3A and DNMT3B, respectively. The N-terminal domain consists of PWWP- and ADD-regulatory domains, six repeats of the CXXC motif, and is followed by the C-terminal catalytic portion [44,49,50,51,52,53]. The PWWP domain has a positively charged surface and can interact with the negative charge of DNA, but is not required for the catalytic activity of de novo DNMT3s [50]. The target recognition domain (TDR) or CXXC is part of the catalytic domain of DNMT3 and is designed to recognize DNA [46]. The slight structural differences between DNMT3A and DNMT3B are key to differential methylation and biochemical interaction with the DNA strand. Specific amino acids are involved in its interaction with the DNA for the conversion of cytosine into 5-metilcytosine. Arg836 of the target recognition domain is essential for the CpG contact by DNMT3A, and Asn779 and Lys777 for CpG recognition by DNMT3B [54,55]. 

### 3.3. Transcriptional and Post-Transcriptional Regulation of DNMTs

**Figure 2:**
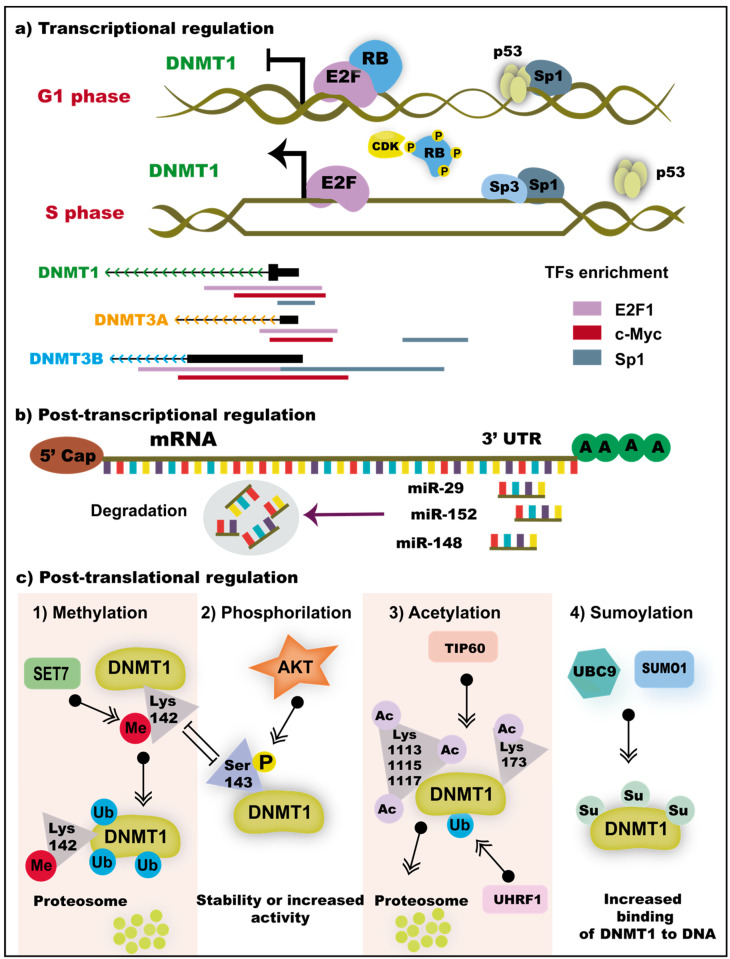
Transcriptional regulation and post-transcriptional modifications of DNMT1. (**a**) The expression of DNMTs increases in the transition from phase G1 to S. In phase G1, p53 interacts with Sp1, and pRB is dephosphorylated and bound to E2F. During replication, the levels of p53 decrease and CDKs expression increases, which results in the dissociation of pRB phosphorylated from E2F and p53 from Sp1; this facilitates DNMT1 transcription activation. DNMT1, DNMT3A, and DNMT3B promoters are enriched with Sp1, E2F, and c-Myc transcription factors (TFs) in several cell lines analyzed in the ENCODE project. (**b**) Post-transcriptional regulation of DNMTs mRNA occurs through the interaction of miRNAs with its 3′ UTR end. The binding of miR-29, miR-152, and miR-148 leads to the degradation of DNMT1 mRNA. (**c**) Post-translational modifications of DNMT1 may favor its activity or degradation. Methylation of lysine 142 by the methyltransferase SET7 leads to the ubiquitination of DNMT1. This modification is mutually exclusive with the phosphorylation of serine 143 by AKT, which favors the stability and activity of DNMT1. The acetylation performed by TIP60 in several lysine residues in combination with the ubiquitination made by UHRF1 leads consequently to the DNMT1 degradation via proteasome. The binding of DNMT1 to DNA can be increased by the sumoylation performed UBC9 and SUMO1. Me, methylation; P, phosphorylation; Ub, ubiquitination; Su, sumoylation; Lys, lysine; Ser, serine.

Both DNMT1 and DNMT3B expression increases during the cell cycle in the transition from G0 or G1 to S phase [56]. Although the regulation of DNMT1 expression has been described in more detail elsewhere, it is proposed that both may be regulated by the same transcription factors (Sp1 and Sp3, Figure 2a). In normal cells, the transcriptional activity of DNMTs promoters is regulated by Sp1, Sp3, E2F, and p53 transcription factors (TFs) binding [57,58,59,60,61,62]. In cells in phase G0 or G1, the Retinoblastoma protein (Rb) is dephosphorylated and binds to E2F resulting in a repression complex on the DNMT1 promoter [60]. This is reinforced by the interaction between Sp1 and p53 in the DNMTs promoter, where p53 functions as a transcriptional repressor by inhibiting the direct binding of Sp1 to the DNMTs promoter [62]. During the S phase, p53 levels are decreased and Sp1 is released, parallel to the decrease in phosphatases and the increase in cyclin-dependent kinases (CDKs) that phosphorylate Rb. Rb phosphorylation promotes the release of E2F, and thus the expression of DNMTs is activated (Figure 2a) [60,61]. Similarly, expression of DNMT3A and DNMT3B depended on Sp1 and Sp3 binding to its promoter, and inhibition of Sp1 or Sp3 binding to their target sites leads to a decreased expression [63].

At the post-transcriptional level, the 3′-UTR of the DNMTs mRNA can be targeted by microRNAs (miRNAs) that recognize and interact with mRNA by base complementarity and contributing to their degradation (Figure 2b) [64,65,66]. For DNMT1 mRNA, the binding of miR-29b [67], miR-152, miR-185 [68], and miR-148 induces its degradation in myeloid leukemia and gliomas cells (Figure 2b). In the presence of miR-16c, miR-222, miR-1741, or miR-1632, DNMT3B expression decreases in ovarian cancer [69]. Similarly, miR-29s is decreased in lung cancer, and its expression inversely correlates with DNMT3A and DNMT3B expression [70]. miR-148 performs a differential regulation on DNMT3B isoform and only downregulates the canonical DNMT3B expression by the binding of its 3′UTR region [71]. Several miRNAs, including miR-26a, miR-26b, miR-26c, miR-203, and miR-222 regulate DNMT3B mRNA in breast cancer cell lines and Burkitt lymphoma [72,73]. The re-expression of these miRNAs reduces the levels of DNMT3B mRNA in hypermethylated breast cancer cell lines [72]. miR-30a-3p is a small noncoding RNA that regulates DNMT3A expression in A549 cells [74].

Post-translational modifications of DNMT1 may favor its stability or lead to their degradation [33]. For example, DNMT1 degradation can be induced by demethylation of lysine 1094 by LSD1 lysine-specific histone demethylase 1A (LSD1) [75]. Similarly, methylation of lysine 142 by SET7 methyltransferase also induces DNMT1 degradation [76]. This last mark is mutually exclusive with serine 143 phosphorylation [77] by AKT1 kinase; when serine 143 is phosphorylated, methylation in lysine 142 is prevented, which prevents the degradation of DNMT1. In some alterations, acetylation can also destabilize DNMT1. For example, Tip60, an acetyltransferase, can acetylate several lysine residues on DNMT1, and subsequently, DNMT1 is ubiquitinated by UHRF1 [78,79]. This mechanism is antagonized by deubiquitinase HAUSP in RKO (cancer) and HEK293 (normal) cells, which protects DNMT1 from proteasomal degradation [79]. Sumoylation is another modification that occurs on DNMT1 and can antagonize the function of ubiquitination. The UBC9 and SUMO1 are proteins that perform the sumoylation of DNMT1 and increase their binding to DNA and their catalytic activity (Figure 2c) [80,81].

## 4. Maintenance of the Methylation Machinery

**Figure 3:**
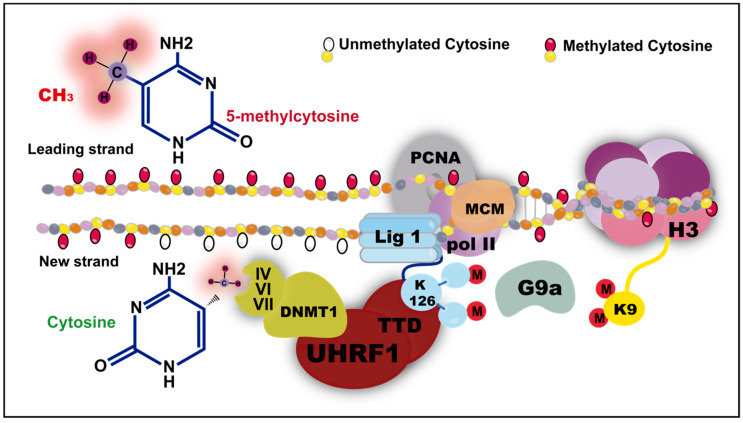
Recruitment of DNMT1 in maintenance methylation. During DNA replication in mammals, the information of the methylation patterns is copied faithfully from the template strand to the nascent strand. The protein complex for localization of DNMT1 is formed by PCNA, which is linked to ligase 1 (Lig 1). Di- or trimethylated ligase 1 in lysine 126 by G9a is tightly bound by the TTD domain of UHRF1, which is consequently linked to DNMT1. The interaction of motif VI of DNMT1 is important to stabilize the DNA–protein interaction. The IV domain of DNMT1 produces a nucleophilic attack on carbon 6, which causes a covalent bond that activates the carbon 5 atom towards the electrophilic attack, and the addition of the methyl group occurs. This is followed by the removal of 1 proton in carbon 5 and the resolution of the covalent interaction to result in the modification of cytosine to 5-methylcytosine.

Although DNMTs directly interact with cytosines in DNA, consensus sequences have not been defined yet. Some proteins participate, directly or indirectly, in the recruitment of DNMT1 to maintain the methylation patterns in each cell division, including PCNA, PAF15, UHRF1, G9a, GLP, and Ligase 1 [82,83]. PCNA is a molecule of the DNA replication complex, first described to interact with DNMT1 in MRC-5 human cells (Medical Research Council cell strain 5). Binding occurs through 163–174 amino acids of DNMT1 and colocalizes with PCNA in the newly replicated DNA replication foci [36]. UHRF has an essential role in maintenance methylation, in embryonic stages of mice. UHRF1 knockout is lethal, and its elimination in mESC leads to the loss of DNA methylation. UHRF1 is a multidomain protein that contains an SET and RING-associated (SRA) domain that binds to hemimethylated DNA [84,85]. UHRF is also frequently bound to K9me2/3 of the tail of histone 3 (H3) through its Tandem Tudor Domain (TTD) [86]. Lysine methylation is a post-translational modification that favors the interaction between proteins. This mark is added by the histone methyltransferases G9a and GLP. Ligase 1 is a canonical protein of the replication complex and has a lysine in a similar motif to H3K9me2/3. Lysine 126 of the ligase 1 behaves physicochemical and structurally like lysine 9 of H3. Both residues (H3K9 and Lig1/K126) can be di- or trimethylated (me2/me3) by G9a and GLP histone methyltransferases, increasing the preference of the UHRF1 TTD by ligase 1 in HeLa cells [82]. Therefore, the location of DNMT1 in the replication foci occurs by the PCNA-LIGASE 1-UHRF1-DNMT1 interaction. This protein complex co-localized in the sites where DNA is synthesized and is responsible for the faithful copy of the information contained in the DNA methylation patterns (Figure 3). The DNMT1 recruitment to maintain the DNA methylation also requires both dual mono-ubiquitylation of PCNA-associated factor 15 (PAF15Ub2) in early S-phase and dual mono-ubiquitylation histone H3 (H3Ub2) by UHRF1 in late S-phase [83]. The puzzle of the maintenance methylation machinery is being solved with recent findings, although there is still a gap in knowledge about methylation of specific genomic sites. Looking deeper, DNMTs, methylation machinery, and *cis* elements are likely to be the answer.

## 5. Elements that Influence DNA Methylation

**Figure 4:**
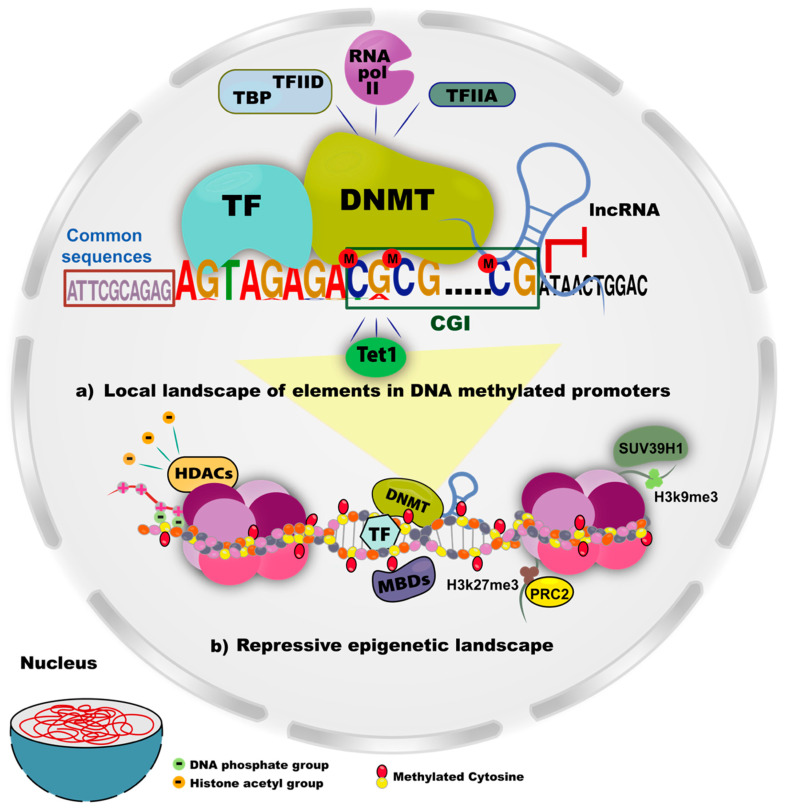
Elements closely related to DNA methylation and repressive epigenetic landscape. (**a**) The DNA methylation deposition in a specific promoter region can be ensured by several mechanisms involving repressors complex and *cis* elements: the presence of CpG islands (CGIs) give the region a propensity for the DNMTs’ enrichment by its dense CpG content. Although little is known about the common motifs present in promoters, they are important for recapitulating specific methylation states in regulatory regions. The DNMTs’ recruitment also occurs by TFs or lncRNAs, resulting in the DNA methylation in the target promoter. These elements, together or separately, regulate the deposition of DNA methylation and prevent active transcriptional machinery and proteins that erased the 5mC patterns (Tet1). (**b**) A wide repressive landscape caused by epigenetics mechanism. Cytosines in CpG context are methylated by *cis* elements influence and DNMTs/partners complex; as a result, this mark is read by repressor proteins such as methylated DNA binding proteins (MBDs) and histone deacetylase (HDACs). HDACs remove acetyl groups from histones to enhance their positive charges and increase the affinity between histones and DNA. In methylated regions, the interaction has been described between EZH2 and DNMTs, resulting in a deposition of the histone repressive mark H3K27me3. In a similar context, the H3K9me3 deposited by SUV39H1 is a frequently found mark in repeat regions that are highly methylated. This landscape is found in inactive chromatin and is a highly repressive level in target genes.

DNMTs and the availability of the substrate S-adenosyl-L-methionine are the limiting factors for the methylation reaction of a CpG site. The location of such effectors is not well known, but there is evidence that DNMTs can be driven by a strong *cis* element or by the sum of several *cis* elements. In both normal and tumor cells, the specific methylation in several genes occurs in conserved DNA sequences as CGI. Additionally, in some genes, the methylation is deposited as a result of the interactions between DNMTs/TFs or DNMTs/lncRNAs. DNA methylation is potentiated by other repressive complexes such as proteins that bind to methylated DNA (Methyl-CpG-binding domain, MBDs), deacetylases of histones (HDACs) that increase the interaction DNA-histones for the chromatin condensation, and histone marks such as H3K27me3 (Figure 4). The functional link of these chromatin regulators with the methylation deposition is described below.

### 5.1. CpGs Content in Promoters

The structural organization of the genome ranges from topological domains to the small motifs of binding for a transcription factor. In this structure’s context, the CGIs are conserved *cis* elements and are a large motif binding for several proteins in genome regulatory regions [11]. DNMTs lack specific binding sequences in the DNA, but they bind to DNA regions depending on their CpG content. CGIs overlap with the transcription initiation site in 60% of human genes and function as platforms of binding sites for TFs and proteins involved in gene transcription. In vertebrates, in a CpG content sense, promoters can be classified into two groups; promoters with a high content of CpGs (HCP) and with low CpGs content (LCP). Both groups are highly conserved in vertebrates [12,87,88]. In general, promoters with low CpG content are targets of both maintenance and de novo methylation, and their genes are repressed transcriptionally [87]. Conversely, promoters with high CpG content have low methylation rates, and they are widely distributed in highly expressed genes. Promoters with intermediate CpGs content are dynamically methylated. Their transcriptional state is regulated via methylation and depends on tissue, differentiation, and cell cycle [12,13,89]. In terms of genomic expression profiles, HCPs are associated with ubiquitously expressed genes, and LCPs were found in genes with specialized functions and expressed in specific cells [90]. Within HCP and LCP promoters, there are regions determining methylation, and these regions are sufficient and necessary in a DNA sequence to recapitulate their hypomethylated or hypermethylated state depending on the cellular context, assuming that they contain the necessary *cis* information to recapitulate their state of natural methylation in pluripotent or differentiated cells [13]. The content of CpGs has been evolutionarily conserved to carefully regulate the switches of gene expression between an embryonic state and a differentiated state. The subtle differences that result in loss of CpGs content are more frequently observed in LCP promoters due to the occurrence of mutations consistent as a result of transitions of methylated cytosines to thymine (5mC < T) [87].

### 5.2. Transcription Factors Involved in DNA Methylation

DNMTs are enzymes that bind to DNA in different regions of the genome, and it is believed that in some cases, enzymes are recruited by tissue-specific TFs that negatively regulate their target genes at promoter level (Table 1). TFs have been described as PML-RAR, whose function is the transcriptional repression of the retinoic acid receptor RARB2, and this occurs through the recruitment of DNMT1 and DNMT3a to the promoter of this receptor [91]. Another example is the TF STAT3, which interacts with DNMT1 and HDAC1 at the SHP-1 phosphatase promoter. SHP-1 is a negative regulator of cell signaling. Therefore, abnormal methylation of SHP-1 in leukemias is, in part, the consequence of DNMT1 recruitment to the promoter by STAT3 [92]. PU.1 is TF and a regulator of hematopoiesis that interacts directly with the ATRX domain of DNMT3A and DNMT3B. PU.1 binding sites gain methylation when DNMT3s are co-expressed with PU.1. One of the best-described targets for PU.1 is the p16INK4A promoter, which is methylated in NIH3T3 cells with overexpression of PU.1, resulting in a decrease in p16INK4A expression. The direct interaction of DNMT3A and DNMT3B was found in the −548 to −2 nt from first p16 ATG and the DNA methylation was sensed in the −480 to 18 of its promoter region [93]. ZHX1 is a zinc finger protein that functions as a TF and interacts with DNMT3B in vivo and in vitro. The N-terminal PWWP domain of DNMT3B is required for its interaction with motifs in the ZHX1 homeobox. Through luciferase assays, it was found that ZHX1 favors the transcriptional repression mediated by DNMT3B in target genes [94]. Through protein arrays for 103 TFs, 42 transcription factors that interact with DNMT3A and DNMT3B were identified, of which 27 interact specifically with DNMT3A and 10 interact exclusively with DNMT3B [95]. In a later work, TFs that interact with DNMT1 were identified. In a TFs array the binding of recombinant DNMT1 (DNMT1R) with 58 transcription factors assayed was found. This analysis confirmed several previously described interactions, such as the DNMT1/Sp1 or DNMT1/p53 interactions, and identified potential interactions not yet described. In situ, proximity ligation assay (P-LISA), Olink/Duolink experiments, and immunoprecipitation confirmed that DNMT1 interacts with Sp1, p53, C-EBPα, and YY1 but not with Sp4 or NFκB-p50 [96]. SALL4 is a stem cell TF that plays a vital role in maintaining the identity of stem cells and controlling self-renewal through transcriptional repression. By means of immunoprecipitation, Western blot, and analysis of the enzymatic activity in HEK293 cells, it was demonstrated that the SALL4 protein interacts directly with different DNA methyltransferases (DNMT1, 3A, 3B, and 3L) and influences the enzymatic activity of the purified DNMTs [97]. The RASSF1A regulation in A549 nonsmall cell lung cancer (NSCLC) cells occurs through the epigenetic silencing. In this context, DNMT3B overexpression is regulated by HOXB3 TF, and physical associations were found between DNMT3B, EZH2, and Myc in the RASSF1A promoter. The recruitment of DNMT3B/EZH2/Myc complex to the RASSF1A promoter is necessary to its epigenetic silencing, in particular, its DNA hypermethylation and consequently improves its function as a tumor suppressor [98]. In Glioma cells, CDKN1a promoter is methylated and decreases its expression as a result of co-recruitment of DNMT3A and Myc [95].

### 5.3. Common Sequences in Methylated Genes

Most CpG dinucleotides in mammals are methylated, but the methylation pattern is not uniform. DNMTs possess an intrinsic preference for particular sequences (Table 1), and this is supported by the notion that methyltransferases evolved from bacterial methyltransferases, which are sequence-specific enzymes [43]. An in vivo study conducted in 2005 determined that DNMT3B prefers the YCpGR sites (Y, pyrimidines; R, purines). These sequences were composed of nucleotides around the central CpG, where the binding affinity of DNMT3B was high for 5′-CTTGCGCAAG-3′ and low for 5′-TGTTCGGTGG-3′ sequences [99]. Sequences adjacent to CpGs are involved in the affinity of human DNMTs for de novo methylation. In the study of de novo motifs on regions commonly methylated by DNMT3B, an enrichment of the T residue in position −1 and residue G in position +1 in the motif found for these genes (NTCpGGN) was observed [100]. This motif was recently confirmed by a biochemical assay, and it was found that DNMT3B specifically recognizes DNA with CpGpG sites [55]. The analysis of the motifs for DNMT3A shows that CpGs are more frequently methylated with a T in position −2 and C in position +2 [101]. In another study, the methylation deposited by DNMT3B was observed on the CANAGCTG (N, any nucleotide) sequence. This sequence was systematically identified in the promoters of the genes methylated by DNMT3B [15]. The contribution of DNMTs to methylation in specific sequences has also been studied into the human hepatocellular carcinoma cell line SMMC-7721. In this context, DNMT1, DNMT3A, and DNMT3B show methylation preferential for particular sequences in the genome. These sequences were found on structural coding genes, repeated DNA sequences, and genes of unknown function. The size of the methylated sequences for DNMT1 was 340 bp, 325 bp for DNMT3A, and 440 bp for DNMT3B. The differential methylation analysis showed 46 fragments exclusively methylated for DNMT1, and their consensus motifs in these fragments were 5′-TAAAAATACAAAAA-3′ and 5′-ATTAGCCGGG-3′, 42 fragments methylated for DNMT3A with consensus motif 5′-TTGCCGGGCT-3′, and 67 fragments methylated for DNMT3B that contain 5′ -GCAGCCGGCAT-3′ motif [100]. In a genome-wide study about protein–DNA interactions (PDIs), a specific binding motif for DNMT3A (CACATCTGGACAGATGTGGGCG) was found to be essential for interaction with DNMT3A [102].

### 5.4. Long Non-Coding RNAs

Long noncoding RNAs (lncRNAs) are part of the 98% of the noncoding genomic DNA [103]. A very important finding after the human genome sequencing was the number of lncRNAs found and their possible functions in gene regulation. According to its structure, the lncRNAs have different functional mechanisms. They can function as scaffolds or guides or interfere with the binding of RNAs and proteins such as DNMTs (Table 1) [104,105]. Genome-wide analysis demonstrated that lncRNAs are deregulated in cell lines and tumors and consequently mediate the overexpression or the recruitment of DNMTs to preferential genomic loci [106,107]. PARTICLE and DACOR1 are lncRNAs that regulate global levels of DNA methylation through regulation of metabolic compounds of the folate pathway and by direct interaction with DNMT1 in HCT116 and MDA-MB-361, respectively [106,108]. Both lncRNAs interact with DNMT1 and are enriched in differently methylated regions (DMRs) on several genes. Furthermore, these lncRNA are involved in the regulation of the DNA methylation cofactor. PARTICLE is implicated in global methylome enhancement through MAT2A (methionine adenosyltransferase 2A gene) methylation. MAT2A participates in synthesis of SAM (S-adenosyl methionine), a key group methyl donor [108]. DACOR1 is downregulated in colon cancer, and its induction in cancer cells results in the decreased expression of genes involved in amino acid metabolism as cystathionine β-synthase (CBS). The reduction of CBS results in the accumulation of homocysteine and an increase in methionine, the substrate needed to generate SAM cofactor [106]. HOTAIR is another lncRNA that regulates global levels of DNMTs. The knockdown of HOTAIR in hepatocellular carcinoma and small cell lung cancer line cells results in decreased expression of DNMT1, DNMT3A, and DNMT3B. The functional consequence is the reduction of methylation on target genes [109,110]. Specifically, there are lncRNAs that influence the recruitment of DNMTs in specific target genes. NEAT1 is an lncRNA overexpressed in osteosarcoma and favors the methylation of E-cadherin through a protein complex with DNMT1, SNAIL, and G9a [17]. LincRNA-p21 interacts with DNMT1 and promotes the methylation of promoters such as Nanog to avoid cell reprogramming [111]. Kcnq1ot1 is another lncRNA that recruits DNMT1 to its target genes in somatic cells [112]. Dum is an lncRNA whose target gene is Dppa2, which is repressed by the *cis* recruitment of DNMT1, DNMT3A, and DNMT3B [16]. There are also lncRNAs that prevent the interaction of DNMTs with the promoters of their target genes. The delicate regulation of the CEBP gene consists of the transcription of its own locus of the lncRNA ecCEBP, which binds to DNMT1 and prevents methylation of the CEBP promoter and genes adjacent to the locus [113]. Another lncRNA with a similar mechanism is DALI. This lncRNA was found to have target genes in *trans* and avoid methylation in its promoters by interaction with DNMT1. The depletion of DALI results in increased methylation of its target genes [114]. The lncRNAs can function as global and specific regulators of DNA methylation by DNMTs increasing expression or recruitment to target genes.

**Table 1:** Elements that favors the interaction and specific targeting for DNMTs.

	DNMT1	DNMT3A	DNMT3B
(a) Transcription factors	STAT3 [92] YY1 [96] Sp1 [96] SALL4 [97] PML-RAR [91] P53 [96]	SALL4 [97] MYC [95] PU.1 [93]	SALL4 [97] PU.1 [93] ZHX1 [94] MYC [98] PML-RAR [91]
(b) Common motifs	5′TAAAAATACAAAAA3′ [100] 5′ATTAGCCGGG3′ [100]	5′TNCpGNC3′ [101] 5′-TTGCCGGGCT3′ [100] 5′CACATCTGGACAGATGTGGGCG3′ [102]	5′CTTGCGCAAG3′ [99] 5′NTCpGGN3′ [101] 5′CpGpG3′ [54] 5′CANAGCTG3′ [13] 5′GCAGCCGGCAT3′ [100]
(c) LncRNA	DALI [114] NEAT1 [15] DACOR [106]. Dum [14] LincRNA-p21 [111] EcCEBP [114] Kcnq1ot1 [112] HOTAIR [109,110] PARTICLE [108]	HOTAIR [109,110] Dum [14]	HOTAIR [109,110] Dum [14]

### 5.5. Histone Methylation Patterns and DNMTs

There are 20 amino acids in histones that can undergo chemical modifications. The histone modifications are positive and negatively correlate with DNA methylation. The association between DNA methylation and histone methylation occurs by the interaction of either DNMTs with histone methylation complex or modified histone residues. The most studied modifications associated with DNA methylation are the trimethylation of lysine 27 in histone 3 (H3K27me3) and lysine 36 in histone 3 (H3K36me3). H3K27me3 is a repressive mark deposited by the PRC2 complex (polycomb repressive complex 2), which is enriched in DNA regions that display abundant DNA methylation and decreased gene transcription. In this context, PRC2 participates in the recruitment of DNMTs [18,115]. Moreover, H3K27me3 is abundant in genes aberrantly methylated in cancer, possibly by the recruitment of de novo methyltransferases [18]. Furthermore, in cells with DNMT3B overexpression, H3K27me3 coexists with DNA methylation in abnormally methylated genes [116]. The methylation of H3K36me3, established by SET, is considered a repressive mark that leads to the distribution of DNA methylation [117]. This mark not only interacts with the PWWP domain of DNMT3A enhancing its activity, but is also necessary for the binding of DNMT3A to the DNA [19]. The trimethylation of lysine 9 in histone 3 (H3K9me3) is a mark that frequently overlaps with pericentromeric regions, sequences highly repeated, regions enriched with heterochromatin, and regions that gain methylation by DNMT3A or DNMT3B. SUV39H is a histone methyltransferase that establishes H3K9me3. Heterochromatin protein 1 (HP1) binds to trimethylated lysine residues to form heterochromatinic subdomains. The SUV39H-HP1 complex and their mark, H3K9me3, are required for DNA methylation by DNMT3A and DNMT3B [118]. Furthermore, H3K9me2/3 is bound by UHRF, a protein essential for maintenance methylation deposited by DNMT1 [83]. Conversely, trimethylation of lysine 4 in histone 3 (H3K4me3) is mutually exclusive with DNA methylation and is enriched in active promoters. When methylated, H3K4 is not recognized by the ADD domain (DNMT3-DNMT3L binding domain) of de novo DNMTs [119]. Temporary histone marks associated with CpG rich genomic regions can be replaced or strengthened by DNA methylation to generate permanent transcriptional repression.

## 6. Perspectives (De Novo DNMTs and Methylation Editing)

Currently, global methylation profiles of most tissues and cells at different stages of development exist [120]. These genome-wide profiles are correlated with RNA expression, histone marks, and nucleosome positioning and determine their positive or negative influence on DNA methylation deposition. However, we are just starting to understand the intricate network of post-translational modifications (chromatin modifications and chromatin factors, including noncoding RNAs) that govern the activity and regulation of DNMTs and their impact on central cellular processes. DNA methylation is a key process in several human diseases, such as cancer and neurological disorders [121,122]. DNA methylation has a key implication in gene function, chromatin biology, cell reprogramming, and medical applications. Differential reprogrammable methylation in a sequence-specific site is now possible through several genome-editing tools based on DNA recognition domains such as transcription activator-like effectors (TALENs), zinc finger proteins (ZNFs), and the system of Clustered Regularly Interspaced Short Palindromic Repeats (CRISPR) and CRISPR-associated (Cas) proteins without genetic editions [123,124,125].

The silencing of specific genes based on DNA methylation is suitable through the fusion of de novo DNMTs’ catalytic domain with catalytically inactive Cas9 (dCas9). dCas9-DNMT is targeted by co-expression of a guide RNA to any 20 bp DNA sequence followed by the NGG trinucleotide present in target genes [125,126]. The fusion dCas9-DNMTs can be guided by multiple sgRNAs to different regions. DNA methylation deposition on promoters impacts their expression and shapes transcription factors binding [127,128]. DNA methylation with dCas9-DNMT3A of IL6ST and BACH2 promoters is heritable across cell divisions and decreases their expression [128]. Novel experimental approaches are necessary to make the CRISPR system more efficient, but it is a very promising tool for epigenetic editing in human diseases.

## 7. Conclusions

The cytosine-methylation patterns in a CpG context are established by three conserved DNA methyltransferases in mammals, and several molecular elements have key roles in specific DNMT localization. DNA methylation contributes to spatiotemporal gene expression regulation. In genes regulated by DNA methylation, particular transcription factors are involved—lncRNAs, CpG content, common motifs, and histone modifications. DNA methylation is influenced in a sequence manner by CpG content, common motifs for DNMTs, and transcription factors. At the chromatin level, histone modifications are positive and negative regulators of DNA methylation. The lncRNAs are elements that regulate DNA methylation by specific interaction between gene, DNMTs, and lncRNA. Deciphering the cytosine methylation code and the underlying basic mechanism will contribute to understanding early developmental stages, proper maintenance of somatic cells, and several human diseases.

## References

[1] Schübeler D. (2015). Function and information content of DNA methylation. Nat. Cell Biol..

[2] Stricker S.H., Köferle A., Beck A.K.S. (2017). From profiles to function in epigenomics. Nat. Rev. Genet..

[3] Lee H.J., Hore T.A., Reik W. (2014). Reprogramming the Methylome: Erasing Memory and Creating Diversity. Cell Stem Cell.

[4] Gruenbaum Y., Cedar H., Razin A. (1982). Substrate and sequence specificity of a eukaryotic DNA methylase. Nat. Cell Biol..

[5] Rhee I., Bachman K.E., Park B.H., Jair K.-W., Yen R.-W.C., Schuebel K.E., Cui H., Feinberg A.P., Lengauer C., Kinzler K.W. (2002). DNMT1 and DNMT3b cooperate to silence genes in human cancer cells. Nat. Cell Biol..

[6] Bestor T.H. (2000). The DNA methyltransferases of mammals. Hum. Mol. Genet..

[7] Riethoven J.-J.M. (2010). Regulatory Regions in DNA: Promoters, Enhancers, Silencers, and Insulators. Methods Mol. Biol..

[8] Angeloni A., Bogdanovic O. (2019). Enhancer DNA methylation: Implications for gene regulation. Essays Biochem..

[9] Wittkopp P.J., Kalay G. (2011). Cis-regulatory elements: Molecular mechanisms and evolutionary processes underlying divergence. Nat. Rev. Genet..

[10] Shibata M., Gulden F.O., Sestan N. (2015). From trans to cis: Transcriptional regulatory networks in neocortical development. Trends Genet..

[11] Gardiner-Garden M., Frommer M. (1987). CpG Islands in vertebrate genomes. J. Mol. Biol..

[12] Saxonov S., Berg P., Brutlag D.L. (2006). A genome-wide analysis of CpG dinucleotides in the human genome distinguishes two distinct classes of promoters. Proc. Natl. Acad. Sci. USA.

[13] Lienert F., Wirbelauer C., Som I., Dean A., Mohn F., Schübeler D. (2011). Identification of genetic elements that autonomously determine DNA methylation states. Nat. Genet..

[14] Deaton A.M., Bird A. (2011). CpG islands and the regulation of transcription. Genes Dev..

[15] Takahashi M., Kamei Y., Ehara T., Yuan X., Suganami T., Takai-Igarashi T., Hatada I., Ogawa Y. (2013). Analysis of DNA methylation change induced by Dnmt3b in mouse hepatocytes. Biochem. Biophys. Res. Commun..

[16] Wang L., Zhao Y., Bao X., Zhu X., Kwok Y.K.-Y., Sun K., Chen X., Huang Y., Jauch R., Esteban M.A. (2015). LncRNA Dum interacts with Dnmts to regulate Dppa2 expression during myogenic differentiation and muscle regeneration. Cell Res..

[17] Li Y., Cheng C. (2018). Long noncoding RNA NEAT1 promotes the metastasis of osteosarcoma via interaction with the G9a-DNMT1-Snail complex. Am. J. Cancer Res..

[18] Schlesinger Y., Straussman R., Keshet I., Farkash S., Hecht M., Zimmerman J., Eden E., Yakhini Z., Ben-Shushan E., Reubinoff B.E. (2006). Polycomb-mediated methylation on Lys27 of histone H3 pre-marks genes for de novo methylation in cancer. Nat. Genet..

[19] Dhayalan A., Rajavelu A., Rathert P., Tamas R., Jurkowska R.Z., Ragozin S., Jeltsch A. (2010). The Dnmt3a PWWP Domain Reads Histone 3 Lysine 36 Trimethylation and Guides DNA Methylation. J. Biol. Chem..

[20] Wheeler H.L. Yale University. Sheffield Scientific School, Johnson TB. Papers on Pyrimidines [Internet]. New Haven, Conn; 1910. 744p. http://archive.org/details/papersonpyrimidi00wheerich.

[21] Johnson T.B., Coghill R.D. (1925). researches on pyrimidines. C111. The discovery of 5-methyl-cytosine in tuberculinic acid, the nucleic acid of the tubercle bacillus. J. Am. Chem. Soc..

[22] Hotchkiss R.D. (1948). The quantitative separation of purines, pyrimidines, and nucleosides by paper chromatography. J. Biol. Chem..

[23] Just T., Waddington C.H. (1939). An Introduction to Modern Genetics. Am. Midl. Nat..

[24] Bestor T.H. (1990). DNA methylation: Evolution of a bacterial immune function into a regulator of gene expression and genome structure in higher eukaryotes. Philos. Trans. R. Soc. Lond. B Biol. Sci..

[25] Dugaiczyk A., Hedgpeth J., Boyer H.W., Goodman H.M. (1974). Physical identity of the SV40 deoxyribonucleic acid sequence recognized by the Eco RI restriction endonuclease and modification methylase. Biochemistry.

[26] Riggs A. (1975). X inactivation, differentiation, and DNA methylation. Cytogenet. Genome Res..

[27] Vanyushin B.F., Tkacheva S.G., Belozersky A.N. (1970). Rare Bases in Animal DNA. Nat. Cell Biol..

[28] Holliday R., Pugh J. (1975). DNA modification mechanisms and gene activity during development. Science.

[29] Van Der Ploeg L.H., Flavell R.A. (1980). DNA methylation in the human γ delta β -globin locus in erythroid and nonerythroid tissues. Cell.

[30] Jones P.A., Taylor S.M. (1980). Cellular differentiation, cytidine analogs and DNA methylation. Cell.

[31] Razin A., Riggs A.D. (1980). DNA methylation and gene function. Science.

[32] Ye F., Kong X., Zhang H., Liu Y., Shao Z., Jin J., Cai Y., Zhang R., Li L., Zhang Y.W. (2018). Biochemical Studies and Molecular Dynamic Simulations Reveal the Molecular Basis of Conformational Changes in DNA Methyltransferase-1. ACS Chem. Biol..

[33] Jeltsch A., Jurkowska R.Z. (2016). Allosteric control of mammalian DNA methyltransferases—A new regulatory paradigm. Nucleic Acids Res..

[34] Pradhan M., Estève P.-O., Chin H.G., Samaranayke M., Kim G.-D., Pradhan S. (2008). CXXC Domain of Human DNMT1 Is Essential for Enzymatic Activity. Biochemistry.

[35] Rountree M.R., Bachman K.E., Baylin S.B. (2000). DNMT1 binds HDAC2 and a new co-repressor, DMAP1, to form a complex at replication foci. Nat. Genet..

[36] Chuang L.S.-H., Ian H.-I., Koh T.-W., Ng H.-H., Xu G., Li B.F.L. (1997). Human DNA-(Cytosine-5) Methyltransferase-PCNA Complex as a Target for p21WAF1. Science.

[37] Berkyurek A.C., Suetake I., Arita K., Takeshita K., Nakagawa A., Shirakawa M., Tajima S. (2014). The DNA Methyltransferase Dnmt1 Directly Interacts with the SET and RING Finger-associated (SRA) Domain of the Multifunctional Protein Uhrf1 to Facilitate Accession of the Catalytic Center to Hemi-methylated DNA. J. Biol. Chem..

[38] Gifford C.A., Ziller M.J., Gu H., Trapnell C., Donaghey J., Tsankov A., Shalek A.K., Kelley D.R., Shishkin A.A., Issner R. (2013). Transcriptional and Epigenetic Dynamics during Specification of Human Embryonic Stem Cells. Cell.

[39] Vilkaitis G., Suetake I., Klimašauskas S., Tajima S. (2004). Processive Methylation of Hemimethylated CpG Sites by Mouse Dnmt1 DNA Methyltransferase. J. Biol. Chem..

[40] Jeltsch A. (2006). On the enzymatic properties of Dnmt1: Specificity, processivity, mechanism of linear diffusion and allosteric regulation of the enzyme. Epigenetics.

[41] Bashtrykov P., Jankevicius G., Smarandache A., Jurkowska R.Z., Ragozin S., Jeltsch A. (2012). Specificity of Dnmt1 for Methylation of Hemimethylated CpG Sites Resides in Its Catalytic Domain. Chem. Biol..

[42] Robertson K.D. (2001). DNA methylation, methyltransferases, and cancer. Oncogene.

[43] Rountree M.R., Bachman K.E., Herman J.G., Baylin S.B. (2001). DNA methylation, chromatin inheritance, and cancer. Oncogene.

[44] Okano M., Xie S., Li E. (1998). Cloning and characterization of a family of novel mammalian DNA (cytosine-5) methyltransferases. Nat. Genet..

[45] Okano M., Bell D.W., Haber D.A., Li E. (1999). DNA Methyltransferases Dnmt3a and Dnmt3b Are Essential for De Novo Methylation and Mammalian Development. Cell.

[46] Gowher H., Loutchanwoot P., Vorobjeva O., Handa V., Jurkowska R.Z., Jurkowski T.P., Jeltsch A. (2006). Mutational Analysis of the Catalytic Domain of the Murine Dnmt3a DNA-(cytosine C5)-methyltransferase. J. Mol. Biol..

[47] Norvil A.B., Petell C.J., Alabdi L., Wu L., Rossie S., Gowher H. (2016). Dnmt3b Methylates DNA by a Noncooperative Mechanism, and Its Activity Is Unaffected by Manipulations at the Predicted Dimer Interface. Biochemistry.

[48] Rinaldi L., Datta D., Serrat J., Morey L., Solanas G., Avgustinova A., Blanco E., Pons J.I., Matallanas D., Von Kriegsheim A. (2016). Dnmt3a and Dnmt3b Associate with Enhancers to Regulate Human Epidermal Stem Cell Homeostasis. Cell Stem Cell.

[49] Qiu C., Sawada K., Zhang X., Cheng X. (2002). The PWWP domain of mammalian DNA methyltransferase Dnmt3b defines a new family of DNA-binding folds. Nat. Genet..

[50] Chen T., Tsujimoto N., Li E. (2004). The PWWP Domain of Dnmt3a and Dnmt3b Is Required for Directing DNA Methylation to the Major Satellite Repeats at Pericentric Heterochromatin. Mol. Cell. Biol..

[51] Jia D., Jurkowska R.Z., Zhang X., Jeltsch A., Cheng X. (2007). Structure of Dnmt3a bound to Dnmt3L suggests a model for de novo DNA methylation. Nat. Cell Biol..

[52] Ishida C., Ura K., Hirao A., Sasaki H., Toyoda A., Sakaki Y., Niwa H., Li E., Kaneda Y. (2003). Genomic organization and promoter analysis of the Dnmt3b gene. Gene.

[53] Xie S., Wang Z., Okano M., Nogami M., Li Y., He W.-W., Okumura K., Li E. (1999). Cloning, expression and chromosome locations of the human DNMT3 gene family. Gene.

[54] Zhang Z.-M., Lu R., Wang P., Yu Y., Chen D.-L., Gao L., Liu S., Ji D., Rothbart S.B., Wang Y. (2018). Structural basis for DNMT3A-mediated de novo DNA methylation. Nat. Cell Biol..

[55] Lin C.-C., Chen Y.-P., Yang W.-Z., Shen J.C.K., Yuan H.S. (2020). Structural insights into CpG-specific DNA methylation by human DNA methyltransferase 3B. Nucleic Acids Res..

[56] Kinney S.R.M., Pradhan S. (2011). Regulation of Expression and Activity of DNA (Cytosine-5) Methyltransferases in Mammalian Cells. Prog. Mol. Biol. Transl. Sci..

[57] Bigey P., Ramchandani S., Theberge J., Araujo F.D., Szyf M. (2000). Transcriptional regulation of the human DNA Methyltransferase (dnmt1) gene. Gene.

[58] Robertson K.D. (2000). Differential mRNA expression of the human DNA methyltransferases (DNMTs) 1, 3a and 3b during the G0/G1 to S phase transition in normal and tumor cells. Nucleic Acids Res..

[59] Margot J.B., Cardoso M.C., Leonhardt H. (2001). Mammalian DNA methyltransferases show different subnuclear distributions. J. Cell. Biochem..

[60] McCabe M.T., Cabioglu N., Summy J., Miller C., Parikh N.U., Sahin A.A., Tuzlali S., Pumiglia K., Gallick G., Price J.E. (2005). Regulation of DNA Methyltransferase 1 by the pRb/E2F1 Pathway. Cancer Res..

[61] Lin R.-K., Wu C.-Y., Chang J.-W., Juan L.-J., Hsu H.-S., Chen C.-Y., Lu Y.Y., Tang Y.A., Yang Y.C., Yang P.C. (2010). Dysregulation of p53/Sp1 control leads to DNA methyltransferase-1 overexpression in lung cancer. Cancer Res..

[62] Peterson E.J., Bögler O., Taylor S.M. (2003). p53-mediated repression of DNA methyltransferase 1 expression by specific DNA binding. Cancer Res..

[63] Jinawath A., Miyake S., Yanagisawa Y., Akiyama Y., Yuasa Y. (2005). Transcriptional regulation of the human DNA methyltransferase 3A and 3B genes by Sp3 and Sp1 zinc finger proteins. Biochem. J..

[64] Braconi C., Huang N., Patel T. (2010). MicroRNA-dependent regulation of DNA methyltransferase-1 and tumor suppressor gene expression by interleukin-6 in human malignant cholangiocytes. Hepatology.

[65] Huang J., Wang Y., Guo Y., Sun S. (2010). Down-regulated microRNA-152 induces aberrant DNA methylation in hepatitis B virus-related hepatocellular carcinoma by targeting DNA methyltransferase. Hepatology.

[66] Zhao S., Wang Y., Liang Y., Zhao M., Long H., Ding S., Yin H., Lu Q. (2011). MicroRNA-126 regulates DNA methylation in CD4+ T cells and contributes to systemic lupus erythematosus by targeting DNA methyltransferase. Arthritis Rheum..

[67] Garzon R., Liu S., Fabbri M., Liu Z., Heaphy C.E., Callegari E., Schwind S., Pang J., Yu J., Muthusamy N. (2009). MicroRNA-29b induces global DNA hypomethylation and tumor suppressor gene reexpression in acute myeloid leukemia by targeting directly DNMT3A and 3B and indirectly DNMT1. Blood.

[68] Zhang Z., Tang H., Wang Z., Zhang B., Liu W., Lu H., Xiao L., Liu X., Wang R., Li X. (2011). MiR-185 Targets the DNA Methyltransferases 1 and Regulates Global DNA Methylation in human glioma. Mol. Cancer.

[69] Lee J.-Y., Jeong W., Lim W., Lim C.-H., Bae S.-M., Kim J., Bazer F.W., Song G. (2013). Hypermethylation and Post-Transcriptional Regulation of DNA Methyltransferases in the Ovarian Carcinomas of the Laying Hen. PLoS ONE.

[70] Fabbri M., Garzon R., Cimmino A., Liu Z., Zanesi N., Callegari E., Liu S., Alder H., Costinean S., Fernandez-Cymering C. (2007). MicroRNA-29 family reverts aberrant methylation in lung cancer by targeting DNA methyltransferases 3A and 3B. Proc. Natl. Acad. Sci. USA.

[71] Duursma A.M., Kedde M., Schrier M., Le Sage C., Agami R. (2008). miR-148 targets human DNMT3b protein coding region. RNA.

[72] Sandhu R., Rivenbark A.G., Coleman W.B. (2012). Loss of post-transcriptional regulation of DNMT3b by microRNAs: A possible molecular mechanism for the hypermethylation defect observed in a subset of breast cancer cell lines. Int. J. Oncol..

[73] Robaina M.C., Mazzoccoli L., Arruda V.O., Reis F.R.D.S., Apa A.G., De Rezende L.M.M., Klumb E.M. (2015). Deregulation of DNMT1, DNMT3B and miR-29s in Burkitt lymphoma suggests novel contribution for disease pathogenesis. Exp. Mol. Pathol..

[74] Wei D., Yu G., Zhao Y. (2019). MicroRNA-30a-3p inhibits the progression of lung cancer via the PI3K/AKT by targeting DNA methyltransferase 3a. OncoTargets Ther..

[75] Wang J., Hevi S., Kurash J.K., Lei H., Gay F., Bajko J., Su H., Sun W., Chang H., Xu G. (2009). The lysine demethylase LSD1 (KDM1) is required for maintenance of global DNA methylation. Nat. Genet..

[76] Estève P.-O., Chin H.G., Benner J., Feehery G.R., Samaranayake M., Horwitz G.A., Jacobsen S.E., Pradhan S. (2009). Regulation of DNMT1 stability through SET7-mediated lysine methylation in mammalian cells. Proc. Natl. Acad. Sci. USA.

[77] Estève P.-O., Chang Y., Samaranayake M., Upadhyay A.K., Horton J.R., Feehery G.R., Cheng X., Pradhan S. (2011). A methylation and phosphorylation switch between an adjacent lysine and serine determines human DNMT1 stability. Nat. Struct. Mol. Biol..

[78] Choudhary C., Kumar C., Gnad F., Nielsen M.L., Rehman M., Walther T.C., Olsen J.V., Mann M. (2009). Lysine Acetylation Targets Protein Complexes and Co-Regulates Major Cellular Functions. Science.

[79] Du Z., Song J., Wang Y., Zhao Y., Guda K., Yang S., Kao H.-Y., Xu Y., Willis J., Markowitz S.D. (2010). DNMT1 Stability Is Regulated by Proteins Coordinating Deubiquitination and Acetylation-Driven Ubiquitination. Sci. Signal..

[80] Lee B., Muller M.T. (2009). SUMOylation enhances DNA methyltransferase 1 activity. Biochem. J..

[81] Melchior F. (2000). SUMO—Nonclassical Ubiquitin. Annu. Rev. Cell Dev. Biol..

[82] Ferry L., Fournier A., Tsusaka T., Adelmant G., Shimazu T., Matano S., Kirsh O., Amouroux R., Dohmae N., Suzuki T. (2017). Methylation of DNA Ligase 1 by G9a/GLP Recruits UHRF1 to Replicating DNA and Regulates DNA Methylation. Mol. Cell.

[83] Nishiyama A., Mulholland C.B., Bultmann S., Kori S., Endo A., Saeki Y., Qin W., Trummer C., Chiba Y., Yokoyama H. (2020). Two distinct modes of DNMT1 recruitment ensure stable maintenance DNA methylation. Nat. Commun..

[84] Sharif J., Muto M., Takebayashi S.-I., Suetake I., Iwamatsu A., Endo T.A., Shinga J., Mizutani-Koseki Y., Toyoda T., Okamura K. (2007). The SRA protein Np95 mediates epigenetic inheritance by recruiting Dnmt1 to methylated DNA. Nat. Cell Biol..

[85] Bostick M., Kim J.K., Estève P.-O., Clark A., Pradhan S., Jacobsen S.E. (2007). UHRF1 Plays a Role in Maintaining DNA Methylation in Mammalian Cells. Sci..

[86] Rothbart S.B., Krajewski K., Nady N., Tempel W., Xue S., Badeaux A.I., Barsyte-Lovejoy D., Martinez J.Y., Bedford M.T., Fuchs S.M. (2012). Association of UHRF1 with methylated H3K9 directs the maintenance of DNA methylation. Nat. Struct. Mol. Biol..

[87] Weber M., Hellmann I., Stadler M.B., Ramos L., Pääbo S., Rebhan M., Schübeler D. (2007). Distribution, silencing potential and evolutionary impact of promoter DNA methylation in the human genome. Nat. Genet..

[88] Elango N., Yi S.V. (2008). DNA Methylation and Structural and Functional Bimodality of Vertebrate Promoters. Mol. Biol. Evol..

[89] Ball M.P., Li J.B., Gao Y., Lee J.-H., LeProust E., Park I.-H., Xie B., Daley G.Q., Church G.M. (2009). Targeted and genome-scale methylomics reveals gene body signatures in human cell lines. Nat. Biotechnol..

[90] Landolin J.M., Johnson D.S., Trinklein N.D., Aldred S.F., Medina C., Shulha H., Weng Z., Myers R.M. (2010). Sequence features that drive human promoter function and tissue specificity. Genome Res..

[91] Di Croce L., Raker V.A., Corsaro M., Fazi F., Fanelli M., Faretta M., Fuks F., Coco F.L., Kouzarides T., Nervi C. (2002). Methyltransferase Recruitment and DNA Hypermethylation of Target Promoters by an Oncogenic Transcription Factor. Science.

[92] Zhang Q., Wang H.Y., Marzec M., Raghunath P.N., Nagasawa T., Wasik M.A. (2005). STAT3- and DNA methyltransferase 1-mediated epigenetic silencing of SHP-1 tyrosine phosphatase tumor suppressor gene in malignant T lymphocytes. Proc. Natl. Acad. Sci. USA.

[93] Suzuki M., Yamada T., KiharaNegishi F., Sakurai T., Hara E., Tenen D.G., Hozumi N., Oikawa T. (2006). Site-specific DNA methylation by a complex of PU.1 and Dnmt3a/b. Oncogene.

[94] Kim S.-H., Park J., Choi M.-C., Kim H.-P., Park J.-H., Jung Y., Lee J.-H., Oh -Y., Im S.-A., Bang Y.-J. (2007). Zinc-fingers and homeoboxes 1 (ZHX1) binds DNA methyltransferase (DNMT) 3B to enhance DNMT3B-mediated transcriptional repression. Biochem. Biophys. Res. Commun..

[95] Hervouet E., Vallette F.M., Cartron P.-F. (2009). Dnmt3/transcription factor interactions as crucial players in targeted DNA methylation. Epigenetics.

[96] Hervouet E., Vallette F.M., Cartron P.-F. (2010). Dnmt1/Transcription Factor Interactions. Genes Cancer.

[97] Yang J., Corsello T.R., Ma Y. (2011). Stem Cell Gene SALL4 Suppresses Transcription through Recruitment of DNA Methyltransferases. J. Biol. Chem..

[98] Palakurthy R.K., Wajapeyee N., Santra M.K., Gazin C., Lin L., Gobeil S., Green M.R. (2009). Epigenetic Silencing of the RASSF1A Tumor Suppressor Gene through HOXB3-Mediated Induction of DNMT3B Expression. Mol. Cell.

[99] Handa V., Jeltsch A. (2005). Profound Flanking Sequence Preference of Dnmt3a and Dnmt3b Mammalian DNA Methyltransferases Shape the Human Epigenome. J. Mol. Biol..

[100] Fan H., Zhao Z., Cheng Y., Cui H., Qiao F., Wang L., Hu J., Wu H., Song W. (2015). Genome-wide profiling of DNA methylation reveals preferred sequences of DNMTs in hepatocellular carcinoma cells. Tumor Biol..

[101] Wienholz B.L., Kareta M.S., Moarefi A.H., Gordon C.A., Ginno P.A., Chédin F. (2010). DNMT3L Modulates Significant and Distinct Flanking Sequence Preference for DNA Methylation by DNMT3A and DNMT3B In Vivo. PLoS Genet..

[102] Hu S., Xie Z., Onishi A., Yu X., Jiang L., Lin J., Rho H., Woodard C., Wang H., Jeong J.-S. (2009). Profiling the Human Protein-DNA Interactome Reveals MAPK1 as a Transcriptional Repressor of Interferon Signalling. Cell.

[103] Djebali S., Davis C.A., Merkel A., Dobin A., Lassmann T., Mortazavi A., Tanzer A., Lagarde J., Lin W., Schlesinger F. (2012). Landscape of transcription in human cells. Nature.

[104] Morris K.V., Mattick J.S. (2014). The rise of regulatory RNA. Nat. Rev. Genet..

[105] Mercer T.R., Mattick J.S. (2013). Structure and function of long noncoding RNAs in epigenetic regulation. Nat. Struct. Mol. Biol..

[106] Merry C.R., Forrest M.E., Sabers J.N., Beard L., Gao X.-H., Hatzoglou M., Jackson M.W., Wang Z., Markowitz S.D., Khalil A.M. (2015). DNMT1-associated long non-coding RNAs regulate global gene expression and DNA methylation in colon cancer. Hum. Mol. Genet..

[107] Schmitz K.-M., Mayer C., Postepska A., Grummt I. (2010). Interaction of noncoding RNA with the rDNA promoter mediates recruitment of DNMT3b and silencing of rRNA genes. Genes Dev..

[108] O’Leary V.B., Hain S., Maugg D., Smida J., Azimzadeh O., Tapio S., Ovsepian S.V., Atkinson M.J. (2017). Long non-coding RNA PARTICLE bridges histone and DNA methylation. Sci. Rep..

[109] Fang S., Gao H., Tong Y., Yang J., Tang R., Niu Y., Li M., Guo L. (2015). Long noncoding RNA-HOTAIR affects chemoresistance by regulating HOXA1 methylation in small cell lung cancer cells. Lab. Investig..

[110] Cheng D., Deng J., Zhang B., He X., Meng Z., Li G., Ye H., Zheng S., Wei L., Deng X. (2018). LncRNA HOTAIR epigenetically suppresses miR-122 expression in hepatocellular carcinoma via DNA methylation. EBioMedicine.

[111] Baoming Q., Wu H., Zhu X., Guo X., Hutchins A.P., Luo Z., Song H., Chen Y., Lai K., Yin M. (2015). The p53-induced lincRNA-p21 derails somatic cell reprogramming by sustaining H3K9me3 and CpG methylation at pluripotency gene promoters. Cell Res..

[112] Mohammad F., Mondal T., Guseva N., Pandey G.K., Kanduri C. (2010). Kcnq1ot1 noncoding RNA mediates transcriptional gene silencing by interacting with Dnmt1. Development.

[113] Di Ruscio A., Ebralidze A.K., Benoukraf T., Amabile G., Goff L.A., Terragni J., Figueroa M.E., Pontes L.L.D.F., Alberich-Jorda M., Zhang P. (2013). DNMT1-interacting RNAs block gene-specific DNA methylation. Nat. Cell Biol..

[114] Chalei V., Sansom S.N., Kong L., Lee S., Montiel J.F., Vance K.W., Ponting C. (2014). The long non-coding RNA Dali is an epigenetic regulator of neural differentiation. eLife.

[115] Viré E., Brenner C., Deplus R., Blanchon L., Fraga M., Didelot C., Morey L., Van Eynde A., Bernard D., Vanderwinden J.-M. (2006). The Polycomb group protein EZH2 directly controls DNA methylation. Nat. Cell Biol..

[116] Zhang Y., Charlton J., Karnik R., Beerman I., Smith Z.D., Gu H., Weiderpass E., Mi X., Clement M.K., Pop R. (2018). Targets and genomic constraints of ectopic Dnmt3b expression. eLife.

[117] Wagner E.J., Carpenter P.B. (2012). Understanding the language of Lys36 methylation at histone H3. Nat. Rev. Mol. Cell Biol..

[118] Lehnertz B., Ueda Y., Derijck A.A., Braunschweig U., Perez-Burgos L., Kubicek S., Chen T., Li E., Jenuwein T., Peters A.H. (2003). Suv39h-Mediated Histone H3 Lysine 9 Methylation Directs DNA Methylation to Major Satellite Repeats at Pericentric Heterochromatin. Curr. Biol..

[119] Ooi S.K.T., Qiu C., Bernstein E., Li K., Jia D., Yang Z., Erdjument-Bromage H., Tempst P., Lin S.-P., Allis C.D. (2007). DNMT3L connects unmethylated lysine 4 of histone H3 to de novo methylation of DNA. Nat. Cell Biol..

[120] Kundaje A., Meuleman W., Ernst J., Bilenky M., Yen A., Heravi-Moussavi A., Kheradpour P., Zhang Z., Wang J., Roadmap Epigenomics Consortium (2015). Integrative analysis of 111 reference human epigenomes. Nat. Cell Biol..

[121] Dawson M.A. (2017). The cancer epigenome: Concepts, challenges, and therapeutic opportunities. Science.

[122] Hwang J.-Y., Aromolaran K.A., Zukin R.S. (2017). The emerging field of epigenetics in neurodegeneration and neuroprotection. Nat. Rev. Neurosci..

[123] Boch J., Scholze H., Schornack S., Landgraf A., Hahn S., Kay S., Lahaye T., Nickstadt A., Bonas U. (2009). Breaking the Code of DNA Binding Specificity of TAL-Type III Effectors. Science.

[124] Pabo C.O., Peisach E., Grant R.A. (2001). Design and Selection of Novel Cys2His2Zinc Finger Proteins. Annu. Rev. Biochem..

[125] Jinek M., Chylinski K., Fonfara I., Hauer M., Doudna J.A., Charpentier E. (2012). A Programmable Dual-RNA-Guided DNA Endonuclease in Adaptive Bacterial Immunity. Science.

[126] Gilbert L.A., Larson M.H., Morsut L., Liu Z., Brar G.A., Torres S.E., Stern-Ginossar N., Brandman O., Whitehead E.H., Doudna J.A. (2013). CRISPR-Mediated Modular RNA-Guided Regulation of Transcription in Eukaryotes. Cell.

[127] Liu X.S., Wu H., Ji X., Stelzer Y., Wu X., Czauderna S., Shu J., Dadon D., Young R.A., Jaenisch R. (2016). Editing DNA Methylation in the Mammalian Genome. Cell.

[128] Vojta A., Dobrinić P., Tadić V., Bočkor L., Korać P., Julg B., Klasić M., Zoldoš V. (2016). Repurposing the CRISPR-Cas9 system for targeted DNA methylation. Nucleic Acids Res..

